# Subacute postoperative myofascial pain diagnosed and treated successfully by ultrasound: a case after laparoscopic hepatectomy

**DOI:** 10.1186/s40981-022-00540-5

**Published:** 2022-07-19

**Authors:** Rumiko Hachisuka, Shima Taguchi, Katsuyuki Moriwaki, Kyoko Oshita, Ayako Umeda, Yasuo M. Tsutsumi

**Affiliations:** 1 Department of Anesthesiology and Pain Medicine, Hiroshima University Hospital, 1-2-3 Kasumi Minami-ku, Hiroshima, 734-8551 Japan; 2 Department of Anesthesiology, Hiroshima Hiramatsu Hospital, 11-27 Hijiyamahonmachi Minami-ku, Hiroshima, 732-0816 Japan; 3 Department of Anesthesiology, JA Hiroshima General Hospital, 1-3-3 Jigozen Hatsukaichi, Hiroshima, 738-8503 Japan

**Keywords:** Postsurgical pain, Myofascial pain, Trigger point injection, Laparoscopic surgery, Subacute pain

## Abstract

**Background:**

Myofascial pain syndrome is one of the causes of prolonged postoperative pain after abdominal surgery. However, diagnosis and treatment of myofascial pain syndrome, especially its myofascial trigger point (MTrP), have not been well established.

**Case presentation:**

A 55-year-old man experienced severe subacute abdominal pain after laparoscopic hepatectomy despite aggressive postoperative pain management. He had a positive Carnett’s sign, indicating abdominal wall pain, 2 weeks after the surgery. Ultrasonography showed a hyperechoic spot surrounded by a hypoechoic area in the inner abdominal oblique muscle under the palpable spot that fulfills the criteria of MTrP. The echogenic MTrP disappeared after repetitive ultrasound-guided trigger point injections (USG TPIs) with pain relief.

**Conclusions:**

Our present case indicates that diagnosing myofascial pain by visualizing the echogenic MTrPs in the abdominal muscles, and subsequent USG TPIs, might provide an accurate maneuver for diagnosis and treatment of subacute myofascial pain after abdominal surgery.

## Background

Myofascial pain syndrome is one of the major etiologies of abdominal wall pain after abdominal surgery [1, 2]. The abdominal wall pain can be diagnosed when the pain fulfills the following criteria: (1) localized pain or a fixed location of tenderness and (2) superficial tenderness or point tenderness or the presence of a Carnett sign [3]. Carnett’s sign means that abdominal pain is provoked by physical abdominal movements, such as bending, straining, and twisting [3, 4]. Aside from myofascial pain syndrome, anterior cutaneous nerve entrapment syndrome is one of the major etiologies of abdominal wall pain [2, 4]. It has been reported that trigger point injections are effective for both nerve entrapment neuropathy and myofascial pain syndrome in the abdominal wall pain [1, 2, 4]. However, the injection target is intramuscular in the case of myofascial pain syndrome [1, 2], while it is perineural space out of muscular in entrapment neuropathy [4]. Ultrasonography might differentiate between these two pathologies. We present here a case where bright (B) mode ultrasound confirmed the presence of a myofascial trigger point in an abdominal muscle in a patient who had subacute postoperative myofascial pain. Written informed consent was obtained from the patient.

## Case description

A 55-year-old man with a height of 160 cm and weight of 65 kg was diagnosed with hepatocellular carcinoma in the liver segment 7 during the patient’s follow-up for hepatitis B and esophageal varices. He underwent laparoscopic hepatic subsegmental resection for 6 h under combined general and epidural anesthesia. Patient-controlled epidural analgesia was introduced postoperatively, and intravenous patient-controlled analgesia with morphine was added to treat refractory abdominal pain. The anesthesiologist’s acute pain service was ended on the fourth postoperative day. The patient’s pain was managed with celecoxib 400 mg per day and on-demand use of loxoprofen 60 mg and tramadol 25 mg. He was discharged on the tenth postoperative day but readmitted because of suspected wound infection. The antibiotics controlled the surgical site infection; however, he suffered from severe recurrent abdominal pain refractory to tramadol and loxoprofen sodium. He was referred to our pain clinic 15 days after surgery.

At the first visit, he complained of an upper abdominal pain rating of 2 at rest and 8 during physical activity on the 11-point Numerical Rating Scale (NRS). He experienced constant aching and posture-induced paroxysmal pain in the right epigastric region. The rotation and lateral bending induced severe pain, indicating positive Carnett’s sign of abdominal wall pain. There was also a muscular hypersensitive tender spot with stiffness compatible with the taut band in the right lateral subcostal area. He felt a pain radiating to the right epigastric region when the tender spot was pressed. Therefore, we diagnosed myofascial trigger point (MTrP) in the tender abdominal muscle. Pregabalin was not effective for muscular pain. After confirming the improvement in inflammatory findings (C-reactive protein level, 1.57 mg/dl) at the second visit, we injected 7 mL of 1% mepivacaine into the MTrP. Immediately after the injection, the pain was relieved.

**Fig. 1:**
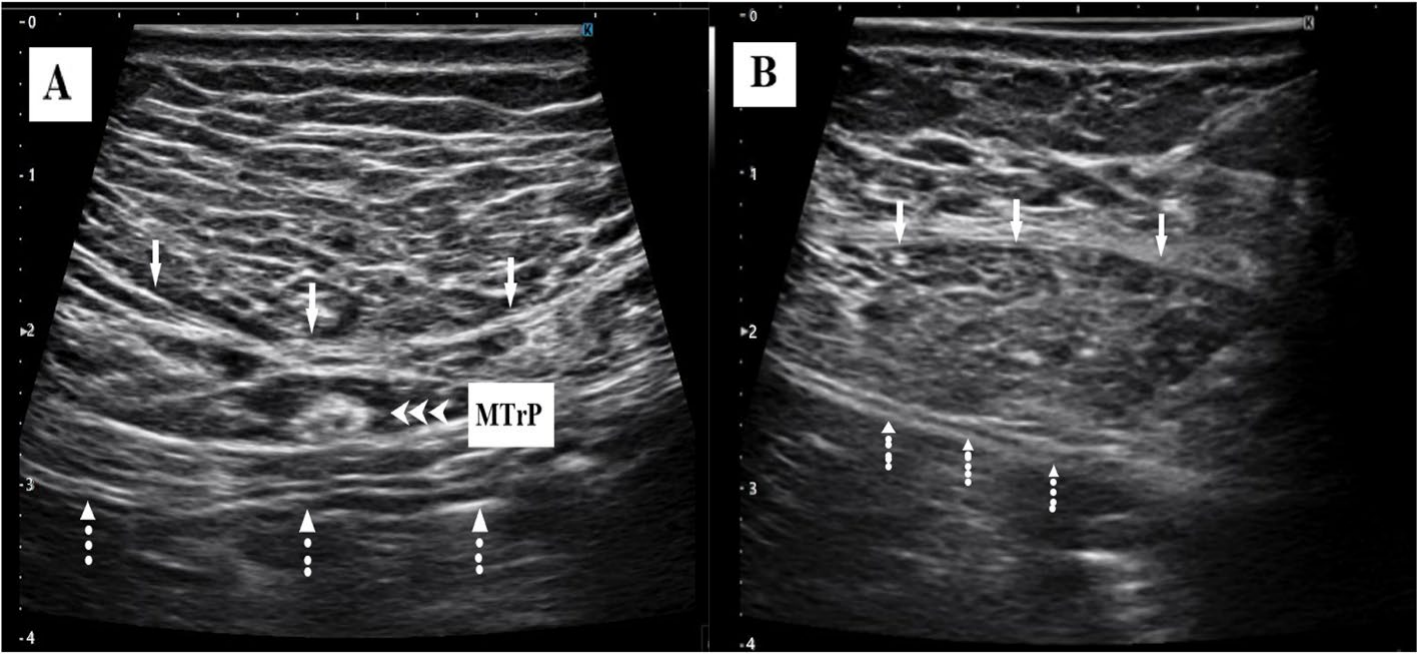
Ultrasound image of the internal oblique abdominal muscle at the second (**A**) and the fifth visit (**B**). A spherical hyperechoic spot with a major axis diameter of 5 mm surrounded by a hypoechoic area is shown at the myofascial trigger point in the internal oblique abdominal muscle (**A**). The hyper- and hypoechoic area disappeared, which changed to an irregular muscle bundle alignment at the same site of **A** and its vicinity (**B**). Vertical arrows indicate the outer fascia of the internal oblique abdominal muscle, and dotted arrows indicate the peritoneum. MTrP, myofascial trigger point

Nineteen days after the first visit, the patient again complained of severe pain with NRS 4 at rest and 8 at physical movement. We decided to apply ultrasound-guided trigger point injection (USG TPI) to the MTrP. The tender spot was located on the lateral side of the right upper abdomen and 5 cm away from the nearest port insertion site. We examined the tender muscle with ultrasound using a linear probe (SONIMAGE HS2TM, 11-3 MHz, Hitachi) and found a hyperechoic spot of 5 mm in diameter surrounded by a hypoechoic area in the internal oblique abdominal muscle (Fig. 1A). The ultrasound image of the muscle surrounding the high echo did not show any loss or abrupt discontinuation of the muscle fibers, which was observed at the port insertion sites. The high echo existed in the internal oblique muscle fibers and seemed different from an entrapped anterior cutaneous nerve echo which should run vertically to the muscle fibers. A 22 G block needle (UNIEVER™ disposable nerve block needle, 70 mm, Unisys, Japan) was proceeded at approximately a 30° angle in the in-plane technique and visualized the needle going into the trigger point. When the tip of the block needle reached the hyperechoic site, the pain was elicited, and the ultrasound showed a local twitch response of the muscle. The pain in the upper abdomen and tenderness disappeared approximately 10 min after injection of 20 mL of 1% mepivacaine to the hyperechoic spot. A total of seven USG TPI sessions were performed once a week.

The hyperechoic spot observed at the third to fourth visit was gradually reduced in size by the treatments. Since the fifth visit, the hyperechoic site disappeared, and the MTrP changed to an irregular muscle bundle alignment with faint echo images (Fig. 1B). The pain gradually decreased, and simultaneously, the degree of muscle stiffness on palpation gradually disappeared. Eventually, the patient’s pain NRS decreased to zero at rest and 1 at physical movement. He felt no pain in his daily life. We ended our treatment on postoperative day 117.

## Discussions

### Diagnosis of MTrP

In this case report, we described a patient with subacute abdominal pain after laparoscopic hepatectomy, whose MTrP was identified by B-mode ultrasonography, and ultrasound-guided trigger point injections (USG TPIs) cured the pain. The diagnosis of myofascial pain requires identifying the presence of MTrP, where the clinical diagnosis of MTrP was made using the international consensus diagnostic criteria for MTrP [5]. According to the criteria, MTrP should fulfill the presence of at least two of the following: a taut band, a hypersensitive spot, or referred pain [5]. Our patient had a pressure-sensitive spot and muscle stiffness compatible with a taut band in the abdominal muscle. The pain also radiated to the epigastric region. These clinical findings led us to diagnose the presence of MTrP. In addition to this clinical diagnosis, we found a hyperechoic spot surrounding the hypoechoic area in the internal oblique muscle at the pain site using B-mode ultrasound. The hyperechoic image was sonomorphologically different from the entrapped nerve echo. When the block needle reached this area, we observed a local twitch response on the echo; at the same time, patient felt a recurrence of severe abdominal pain. A local twitch response, defined as a phenomenon in which muscle contraction occurs when pressure stimulation is applied to MTrP, is a helpful finding to diagnose myofascial pain [6]. Two reports have shown that ultrasonography can be used to identify local twitch responses in deeper muscles [7, 8]. We, therefore, identified the intramuscular echogenic area as MTrP. The disappearance of the hyperechoic and hypoechoic area by repeated USG TPIs and relief of pain support the diagnosis that the patient’s abdominal wall pain was due to myofascial pain caused by MTrP formation.

### Echogenic MTrP

US imaging via bright (B) mode, Doppler, or elastography has been previously reported to provide objective evidence of MTrPs by identifying areas with echo images distinct from normal tissue [6, 9, 10]. In this case report, we found a 5-mm-diameter hyperechoic spot surrounded by a hypoechoic area in the internal oblique abdominal muscle in B-mode ultrasound. Both hypoechoic and hyperechoic findings have previously been reported separately [9, 11]. Three case reports documented a hyperechoic area [11–13]. One reported hyperechoic area of a diameter of 5 mm in close contact with the superficial fascia of the muscle [12]. Other reports showed a cotton ball-like or a plate-like hyperechoic image [11, 13]. The hyperechoic image at the MTrP may reflect pathological changes in the muscle fibers [13]. On the other hand, spherical/elliptical or banded-shaped and dark gray hypoechoic images have been reported [9, 14]. Shah et al. have demonstrated that MTrP had high concentrations of inflammatory neuropeptides such as substance P and calcitonin gene-related peptide [6]. The hypoechoic region may reflect the accumulation of tissue fluid associated with this inflammatory response. Because the hyper and hypoechoic MTrP were previously reported separately, this is the first report that demonstrated the simultaneous presence of both the hyper and hypoechoic areas.

### Limitations

Although our case showed a positive correlation between pain relief and disappearance of the abnormal intramuscular echogenic MTrP and successfully differentiated MTrP from entrapment neuropathy, the other possible causes of postoperative abdominal wall pains, such as hernia [1] and nerve injury-related neuropathic pain [15], should also be investigated as differential diagnoses. In a recent systematic review on ultrasound-guided interventional procedures for MTrP, Diep et al. described that despite diagnostic ultrasound imaging being a promising method that can be used to strengthen the reliability of MTrP localization, the result of their systematic review showed that the value of US-guided interventions remains unclear for treatment of myofascial pain syndrome [10]. Further research is required to evaluate the efficacy of USG TPIs in patients with postoperative abdominal wall pain.

## Conclusion

Our case report indicates that subacute pain after abdominal surgery may originate from myofascial pain in some patients, and myofascial pain might be diagnosed objectively by B-mode ultrasound and treated with USG TPI. Diagnosis and treatment of MTrP using ultrasound may help prevent the transition from subacute to chronic pain in postoperative abdominal myofascial pain patients.

## Data Availability

Data relevant to this case report are not available for public access because of patient privacy concerns but are available from the corresponding author on reasonable request.

## Abbreviations

MTrP: Myofascial trigger point
NRS: Numerical Rating Scale
USG TPI: Ultrasound-guided trigger point injection
